# Perceptions of Pacific children’s academic performance at age 6 years: A multi-informant agreement study

**DOI:** 10.1371/journal.pone.0240901

**Published:** 2020-10-16

**Authors:** Hyun Min Kim, Brigid McNeill, John Everatt, Leali’ie’e T. Taleni, El-Shadan Tautolo, Gail Gillon, Philip J. Schluter

**Affiliations:** 1 School of Health Sciences and Child Wellbeing Research Institute, University of Canterbury–Te Whare Wānanga o Waitaha, Christchurch, New Zealand; 2 School of Teacher Education and Child Wellbeing Research Institute, University of Canterbury–Te Whare Wānanga o Waitaha, Christchurch, New Zealand; 3 Faculty of Health and Environmental Sciences, Auckland University of Technology, Auckland, New Zealand; 4 School of Clinical Medicine, Primary Care and Clinical Unit, University of Queensland, Brisbane, Australia; University of Macau, MACAO

## Abstract

**Purpose:**

In New Zealand, Pacific immigrants are among the fastest growing ethnic minorities but, as a group, they are also at most risk of not realising their literacy and educational aspirations critical for achieving their human potential and wellbeing. This may be due, in part, to a misalignment in the shared understanding of academic success between students, parents and their teachers within largely non-Pacific school environments. This study aims to report levels of agreement in child-mother, child-teacher, and mother-teacher perceptions of Pacific children’s academic performance at age 6 years.

**Method:**

A cohort of Pacific infants born during 2000 in Auckland, New Zealand, was followed as part of the Pacific Islands Families study. Maternal home interviews were conducted at 6-weeks and 6-years postpartum, together with separate child and teacher elicitations at 6-years. Pairwise agreement of academic performance responses was assessed using Cohen’s weighted κ statistic, along with symmetry and marginal homogeneity tests.

**Results:**

At 6-years, information was available for 1,001 children and their mothers, and teachers’ evaluations for 549 children. Negligible to slight agreements and significant asymmetry were found between the child-mother (κ = 0.03, 95% CI: -0.03, 0.09), child-teacher (κ = 0.04, 95% CI: 0.01, 0.08), and mother-teacher (κ = 0.07, 95% CI: 0.03, 0.11) pairwise assessments–with children and mothers more likely to rate Pacific children’s academic performance higher than their teachers. Significantly higher concordances with teacher assessments were found among mothers with post-secondary education, proficiency in English, and stronger alignment with New Zealand culture and for children who performed strongly on a standardised measure of performance relative to their peers.

**Conclusion:**

Strategies are needed to align Pacific students’ and parental perceptions with documented educational achievement outcomes and to facilitate more effective and timely feedback on achievement results and home-school communication. The importance of removing language, cultural and socio-economic barriers to achieving shared understanding of academic performance between teachers and families is highlighted.

## Introduction

Education is a vital catalyst for child development and the realisation of human potential and wellbeing. Indeed, the 2015 Incheon Declaration confirms that education develops the skills, values and attitudes that enable citizens to lead healthy and fulfilled lives, make informed decisions, and respond to local and global challenges [1]. Despite this, educational achievement and its bi-directional connection to health have received relatively scant attention within the domain of population health research [2–4]. Language and literacy acquisition precedes most other children’s educational development, and successful early attainment of these skills is essential for later educational achievement [5]. However, within New Zealand, a developed and relatively wealthy nation, one in five children struggle with learning to read [6]. Moreover, the nation’s disparity between children’s high and low reading and numeracy performance is one of the largest in the developed world [6]. Children of Māori and Pacific ethnic identities, and those of lower socioeconomic positions, are over-represented in these poor statistics [6, 7]. The current situation is unacceptable to many (see for example, [8]), including the Ministry of Education–te Tāhuhu o te Mātauranga (MoE) which has a stated purpose to deliver equitable outcomes and help every New Zealander achieve excellence and be strong in their national and cultural identity [9].

Pacific people in New Zealand, who comprised 8.1% of the total New Zealand population at the 2018 Census, are relatively young ethnic minorities with median age of 23.4 years compared to the dominant New Zealand European/Pākehā population who constituted 70.2% of the population and had median age of 41.4 years [10]. Pacific people’s cultural heritage is diverse and manifests in many differing traditions, languages, and histories of immigration [11]. Their cultural values and adherence to traditions importantly shape their views on education, health and wellbeing [12, 13]. For most Pacific communities, health is not confined to the physical domain but is an embodiment of physical, mental, social and spiritual domains that indicates a general state of wellbeing at both individual and community levels [13]. Pacific students’ cultural identities are also reflected in their educational goals and expectations [8, 12, 14]. The value of education to Pacific people is central, indeed it often forms part of the *raison d’etre* for immigration, yet New Zealand’s inequitable and poor statistics belie this, and may represent different expectations or understandings of success [15].

Within New Zealand, education, and literacy in particular, has been predominantly defined by its New Zealand European/Pākehā population, and is largely provided in monolingual English [7]. By contrast, many Pacific people (including Māori, New Zealand’s indigenous people) originate from oral language traditions that emphasised other forms of literacy such as the spoken word, stories, metaphors, proverbs, visuals arts, dance and song–together with their own written language [7, 16]. The level of discord in perceptions of literacy achievement held between Pacific and New Zealand European/Pākehā populations is unknown, yet it may importantly contribute to the disparity in New Zealand children’s documented reading performance [6, 7]. The impact of discordance would be particularly marked if perceptions of language and literacy achievements and markers of educational success differ between these ethnic groups, teachers and the measured benchmarks.

To date, limited attention has been paid to studying the extent of agreement in parent, child and teacher perceptions of early language and literacy success in the general populations. Therefore, if we expand our search and draw from the extant evidence from the studies of children with known behavioural and learning difficulties (LDs), the value of multi-informant assessments and the analyses of concordances between those assessments is seldom disputed [17, 18]. For example, in a 1994 study of students with LDs aged between 11 to 16 years, slight to moderate agreements in perceptions of academic performance were found between parent-child, child-teacher, and parent-teacher dyads [19]. Other studies have focused on agreement across the speech-language assessment (SLA), teacher and family assessments of language ability and/or language intervention need rather than academic success [20–22]. A 2006 study of 60 pre-school aged children compared teacher judgement with standardised measures of general language and phonological awareness [20]. Although judgements were significantly related, sensitivity and specificity were considered less than acceptable (estimated at 86% and 68%, respectively) [20]. Similarly, in 2008, Massa and colleagues reported moderate concordance rates between parent and teacher observational rating scales and standardised language and literacy assessment [21].

More recently, in a 2017 study involving 157 children aged between 4 and 5 years, formal SLA and teacher and parent report of identification of speech sound disorder were compared [22]. Higher rates of concordance (86–90%) were reported for parent-SLA than for teacher-SLA (63–80%) [22]. The authors attributed low concordance between SLAs and teachers to state policy regarding eligibility for speech therapy which was generally focused around children with most significant intervention need. Other factors that may have underpinned discordance across groups were not explored. Future work that focuses on larger samples of children and relates SLA to parent and teacher ratings of academic success is also necessary as we look towards a model of greater collaboration and partnerships between teachers and families to help children achieve functional goals.

Although some attributed the lack of concordance among different informants to response unreliability, others have argued that they reflect valid contributions of different information [17, 21–23]. Indeed, pooling information from multiple informants and analysing differences can help researchers to obtain a more comprehensive picture with which to predict children’s behaviours or diagnose LDs [17, 18, 24, 25]. While the studies reviewed above help elucidate the level of shared understanding between families and teachers about their children with known LDs, to our knowledge, no studies have quantified the extent of concordance among the general population of Pacific children, mothers and teachers.

Academic self-concept can be linked to one’s perceptions and attitudes toward their own ability, competence and proficiency in broad areas of academic tasks [26]. Academic self-concept, like other domains of self-concept is generally considered to be a multidimensional construct and to have a reciprocal relationship with academic achievement [26–28]. In a longitudinal study of 60 children aged between 5 and 8 years, the children with positive academic self-concept performed more strongly on reading related tasks than did the children with negative or typical academic self-concept [26]. The study also demonstrated that academic self-concept could influence academic achievement much earlier than previously hypothesised. It is thus important to investigate Pacific children’s academic self-concept and how it may relate to the documented assessment results in their early years at school.

Some have noted that the primary basis often cited by researchers for not including children’s perspectives is the lack of reliability and validity in children’s self-reported measures [29]. However, this stance conflicts with the peer- and self-assessment (PASA) strategies promoted by those who support using learning assessments primarily for formative purposes [30–32]. The proponents of formative learning practices emphasise the benefits of PASA in empowering students, developing students’ self-regulation and communication skills, and improving student engagement [33–35]. A synthesis of students’ and teachers’ perspectives on PASA practices observed in New Zealand classrooms conducted in 2013 revealed that students and teachers placed different emphases across the main conceptions of assessment [32]. This may explain the low to moderate concordances between students’ self-assessment and teachers’ evaluations or test performances found in studies reviewed by Brown and colleagues in 2015 [34]. However, the relationship between differing perceptions arising from cultural differences and the impact on children’s academic performance remains largely unknown. This study explicitly investigates children’s perspectives on their school performance in the first year of school.

General population information on perceptions of children’s early academic performance within New Zealand has not been routinely collected. However, the Pacific Islands Families (PIF) study, a birth cohort study of 1,398 Pacific children growing up in New Zealand [11], has elicited perceptions of early academic performance held by Pacific children aged 6 years, their mothers, and teachers. All children in New Zealand are legally required to have started their formal education by age 6 years. However, most start their primary school education on their 5^th^ birthday, which means that they would have had approximately one year of formal instruction by the age 6 years. The availability of teacher’s overall assessment of children’s literacy and numeracy skills at this age allows the researchers to study the outcomes of children’s early instruction. Therefore, Pacific children’s self-reported academic performance at this age can be obtained from the 6-years measurement wave of the PIF study and contrasted with the responses from the children’s mothers and teachers. This enables the empirical investigation into the concordance or discordance of these perceptions among a general Pacific population.

Accordingly, the primary aim of this study is to report levels of agreement and symmetry in child-mother, child-teacher, and mother-teacher perceptions of Pacific children’s academic performance. A secondary aim is to investigate purposefully selected characteristics that differ between concordant child-teacher and mother-teacher pairs and their discordant pair counterparts. This aim seeks to examine any socio-demographic and cultural differences between concordant and discordant pairs and hence analyse any association between those differences and the observed patterns of concordance. Another secondary aim is to relate teachers’ assessments of children’s oral language skills to the students’ performance in a psychometrically robust, direct measure of receptive vocabulary skills, the British Picture Vocabulary Scale (BPVS) [36]. This latter aim is used to establish the reliability of teachers’ assessments, as an educational achievement reference standard.

## Methods

### Study design

A cross-sectional agreement study nested within the PIF birth cohort study was conducted.

### Participants

The PIF study follows a birth cohort of Pacific children born in 2000, and incorporates data elicited from these children, their mothers and teachers.

### Procedure

A detailed account of the PIF study design, methods, instruments and its procedures has been provided previously in cohort publications [11, 37]. In short, the interest for participation in the study was garnered from mothers due to give birth at Middlemore Hospital, South Auckland, and all potential PIF cohort were selected at birth between 15 March and 17 December 2000. The key recruitment criteria were that infant had at least one parent who identified as being of Pacific ethnicity and a New Zealand permanent resident. Recruitment was facilitated through the hospital Birthing Unit, with help from the Pacific Islands Cultural Resource Unit, and consent was sought from mothers to make a home visit within 6 weeks of the childbirths. Around 6-weeks postpartum, female Pacific interviewers fluent in both English and Pacific language(s) visited potential participants at home to confirm study eligibility and obtain informed consent. Once written consent was obtained, mothers were asked to participate in one-hour interviews concerning family’s socioeconomic functioning, maternal health and the infant development. Home visits were repeated approximately 1-year, 2-years, 4-years, and 6-years postpartum. With maternal consent and child assent, the children were independently interviewed for the first time at the 6-years measurement wave, mostly within their school settings. Also at this time, the children’s teachers were approached to participate in the study.

### Measures

#### Standardised measurement

The BPVS was used to measure children’s receptive vocabulary skills at 6-years [36]. It is a standardised test where participants are asked to select the appropriate answer from four possible pictures. Word-items get progressively more difficult until each child’s limit is reached. Entries cover a wide range of language levels as well as word classes and are allocated to different semantic and/or grammatical groupings (e.g. actions, adjectives, animals and parts, books, human body parts, buildings, emotions and social expression, food). The test consists of 14 question sets, with each set containing 12 vocabulary entries. Here, raw scores were age-standardised employing the British population norm [36]; see S1 Table for more information.

#### Self-reported assessment of children’s academic performance

Mothers, teachers and children were asked to evaluate the children’s academic performance at 6-years using 5-point Likert-type scales. Children responded to the statement “I am good at school work”, with options: yes always (C5); yes sometimes (C4); neither yes or no (C3); no sometimes (C2); and, no always (C1). Mothers provided response to the question “Based on your knowledge of your child’s school work, how is he/she doing in school this year?”, with response options: very well (M5); well (M4); average (M3); poorly (M2); and, very poorly (M1). Teachers were asked to separately evaluate the children’s performance in reading, oral language, written language and mathematics domains. Response options used the scale: excellent (5); very good (4); satisfactory (3); needs improvement (2); and, very poor (1). To form a global score assessment, these four scores were summed and categorised into five levels using the derived mean ($\bar{x}$) and standard deviation (SD), via: [$\bar{x}$ + 1.5×SD, 20] (T5); [$\bar{x}$ + 0.5×SD, $\bar{x}$ + 1.5×SD) (T4); [$\bar{x}$ – 0.5×SD, $\bar{x}$ + 0.5×SD) (T3); [$\bar{x}$ – 1.5×SD, $\bar{x}$ – 0.5×SD) (T2); and, [4, $\bar{x}$ –1.5×SD) (T1). In an additional sensitivity analysis, the global teacher assessment scores were recreated excluding the assessment of children’s performance in mathematics–to improve alignment with the standardised measure of linguistic abilities used–and reanalysed.

### Sociodemographics

Maternal information at baseline (the 6-weeks measurement wave) included age, ethnic identification, highest educational attainment, parity, relationship status, English language fluency, cultural orientation, and household income (see S2 Table for more information on selected variables). Cultural orientation was conceived using Berry’s bi-directional framework [38] and measured via an adapted General Ethnicity Questionnaire (GEQ) [39] tailored to the New Zealand and Pacific cultural settings [15]. Mothers were categorised into one of the following four orientation types: integration (strong alignment with both cultures); assimilation (strong New Zealand, weak Pacific); separation (weak New Zealand, strong Pacific); and marginalisation (weak alignment with both cultures) [15]. The modified GEQ included items that were related to Pacific mothers’ social connections, languages and access to cultural resources and ties [15]. The bi-directional framework posits that the host and migrant cultures interact and influence each other [38]. Within this framework, one’s cultural orientation is viewed as being on a continuum between the two cultures, and the individual’s position is constructed and analysed in relation to those of their peers [38].

Child information included sex, ethnic identification, and internalising/externalising problem behaviours. For the latter, mothers were asked to complete the Child Behaviour Checklist (CBCL) for children aged 6–18 years [40] to assess internalising (depressed/withdrawn) and externalising (aggressive/ disruptive) problem behaviours; see S2 Table [41].

Teacher information included sex, ethnic identification and years of teaching experience. The teachers were asked to disclose all ethnic groups that they identified with and to select the one that they identified with the most. Preference was given to the latter information.

### Statistical analysis

Reporting of analyses was informed by the STROBE guidelines for observational studies [42] and the GRRAS guidelines for reporting reliability and agreement studies [43]. Initially, data checks were undertaken, and then participant flow and descriptive statistics were reported. To avoid within-family collinearity bias, one child from all twin births was randomly removed. Patterns of missing data for child and maternal characteristics between those with and without a teacher’s assessment were evaluated using Fisher’s exact test [44]. Participants’ pairwise response distributions were assessed using McNemar’s test of symmetry [45] and Stuart-Maxwell’s test of marginal homogeneity [46], and inter-rater agreement was assessed using Cohen’s weighted kappa (κ) statistics with the square function penalty [47]. The computed κ statistics were interpreted using Landis and Koch’s approach where 0≤κ≤0.2 represents negligible to slight agreement; 0.2<κ≤0.6 represents fair to moderate agreement; and 0.6<κ represents strong agreement [48]. Teacher assessment of children’s oral language skills was compared to the children’s performances in the BPVS raw scores using unadjusted and adjusted ordinal logistic regression models fitted using the proportional odds assumption. As a form of sensitivity analysis, the concordance and agreement analyses are repeated with the global teacher assessment scores comprising only of the language and literacy domains. Next, Fisher’s exact test was employed to investigate the patterns of child-teacher and mother-teacher concordance and discordance over the considered sociodemographic variables [44]. Potential confounding between the maternal cultural orientation measure and the demographic variables that were associated with the concordance patterns were further explored. Analyses were performed using R version 3.6.1 (The R Foundation, Vienna, AT), SAS version 9.4 (SAS Institute Inc., Cary, NC, USA) and Stata SE version 15.1 (StataCorp, College Station, TX, USA), and α = 0.05 defined statistical significance.

### Ethics

Ethical approval for the PIF study was obtained from the Auckland Branch of the National Ethics Committee and the Health and Disability Ethics Committee. In addition to the routine ethics approval procedures, the PIF measures and assessment tools have been reviewed by a select group of Pacific scientists and community leaders and piloted prior to implementation [11, 15]. The PIF study adheres to the guidance stipulated under the Helsinki Declaration for human experimentation [11].

## Results

### Participants

At baseline (6-weeks postpartum), 1,376 mothers gave consent to participate in the PIF study, and the information on those mothers and their 1,398 children (22 twins) were collected [11]. The mean maternal age at childbirth was 27.9 years (range: 14, 57 years), with 8% of them being younger than 20 years, while 4% were older than 40. About 28% of the children were their mothers’ first-born [11]. Overall, 1,107 (80.5%) mothers were partnered, 454 (33.0%) were New Zealand-born, and 535 (38.9%) had no formal educational qualifications [11]. At the 6-years measurement wave, 1,001 maternal and 1,019 child interviews were completed (retention rate of 72.7% and 72.9%, respectively). Randomly removing a child from each multiple birth left 1,001 child-mother pairs. Teachers’ information was available for 549 (54.8%) of these children, along with their evaluations for the children’s performance in at least one of the four academic areas: reading, writing, oral language, and mathematics. Characteristics of the participants are summarised in Table 1.

**Table 1:** Distribution of the participating children’s (n = 1,001), mothers’ (n = 1,001), and teachers’ (n = 549) characteristics.

		n	(%)
**Child characteristics**			
*Sex*			
	Female	485	(48.5)
	Male	516	(52.5)
*Ethnic identification*^a^			
	Samoan	463	(51.3)
	Tongan	174	(19.3)
	Cook Islands Māori	47	(5.2)
	Other Pacific	218	(24.2)
*CBCL internalising behaviour*^b^			
	Normal range	841	(84.1)
	Borderline	75	(7.5)
	Clinical	84	(8.4)
*CBCL externalising behaviour*^b^			
	Normal range	709	(70.9)
	Borderline	145	(14.5)
	Clinical	146	(14.6)
**Maternal characteristics at baseline**			
*Age at childbirth (years)*^b^			
	<20	73	(7.3)
	20–24	241	(24.1)
	25–29	278	(27.8)
	30–34	229	(22.9)
	35–39	143	(14.3)
	≥40	36	(3.6)
*Ethnic identification*			
	Samoan	463	(46.3)
	Tongan	218	(21.8)
	Cook Islands Māori	174	(17.4)
	Other Pacific†	78	(7.8)
	Non-Pacific	68	(6.8)
*Highest educational qualification*			
	No formal qualification	371	(37.1)
	Secondary school	358	(35.8)
	Post-secondary school	272	(27.2)
*Parity*^c^			
	1	251	(25.5)
	2–4	561	(57.0)
	≥5	172	(17.5)
*Relationship status*			
	Married/de facto	814	(81.3)
	Single	187	(18.7)
*English fluency*			
	Proficient	626	(62.5)
	Otherwise	375	(37.5)
*Cultural orientation*^d^			
	Integration (High Pacific/High NZ)	183	(18.5)
	Assimilation (Low Pacific/High NZ)	316	(31.9)
	Separation (High Pacific/Low NZ)	314	(31.7)
	Marginalisation (Low Pacific/Low NZ)	178	(18.0)
*Household income (New Zealand dollars)*			
	≤$20,000	331	(33.1)
	$20,001-$40,000	511	(51.0)
	>40,000	120	(12.0)
	Unknown/declined to answer	39	(3.9)
**Teacher characteristics at 6-years**			
*Sex*^e^			
	Female	515	(94.7)
	Male	29	(5.3)
*Ethnic identification*^f^			
	New Zealand European/Pākehā	262	(48.3)
	Pacific	95	(17.5)
	Māori	27	(5.0)
	Other	158	(29.2)
*Years of teaching experience*^g^			
	0–5	256	(47.9)
	6–10	126	(23.6)
	>10	153	(28.6)

^a^99 (9.9%) values missing

^b^1 (0.1%) value missing

^c^17 (1.7%) values missing

^d^10 (1.0%) values missing

^e^5 (0.9%) values missing

^f^7 (1.3%) values missing

^g^14 (2.6%) values missing.

† Includes those who identified with multiple Pacific ethnic groups.

The availability of teacher assessment was not related to any of the child and maternal characteristics presented in Table 1 (all Fisher’s exact test p>0.05), except for maternal education (Fisher’s exact test p = 0.001) and parity (Fisher’s exact test p = 0.03). Children without a teacher’s assessment were more likely to have mothers without any formal qualifications (43.1% vs. 32.1%) and parity of 1 (27.6% vs. 23.8%) or ≥5 (19.9% vs. 15.5%) than children with an assessment.

### Assessments of children’s academic performance

**Fig 1:**
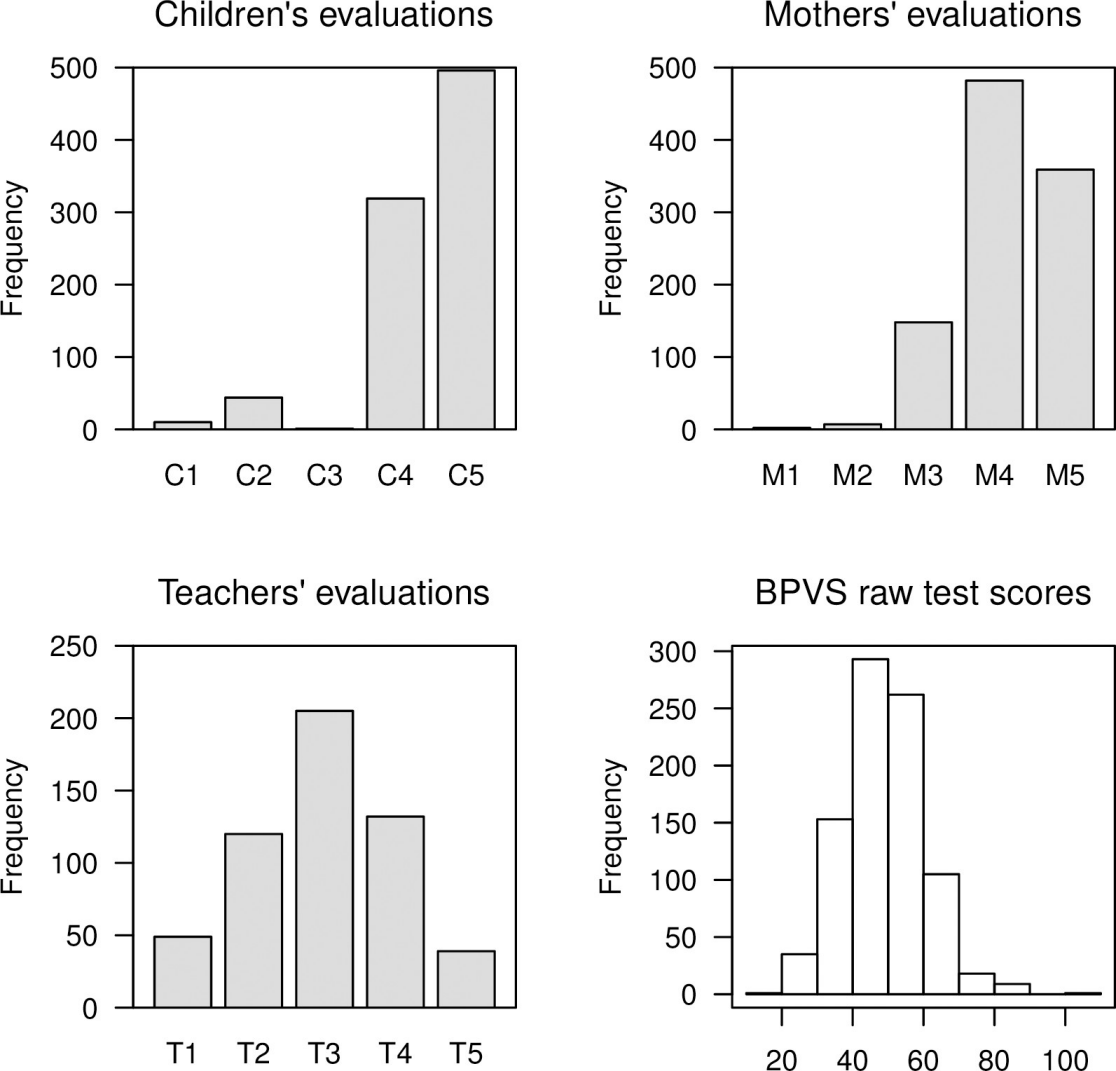
Perceptions of Pacific children’s academic performance at age 6 years. Each participant group rated the children’s academic performance using a Likert-type scale from 1 to 5 with 5 being the highest level of achievement.

Complete self-assessment data were available from 870 (86.9%) children, together with 998 (99.7%) maternal responses and 545 (99.3%) teacher global measures. Fig 1 presents the distribution of these responses, together with a histogram of the BPVS raw scores, available from 877 (88.6%) children. At age 6 years, the BPVS raw scores ranged between 18 and 104 with mean score of 49.4 (SD = 11.4). Fig 1 reveals that the teachers’ global assessment distribution is largely symmetrical, whereas both the children’s and maternal distributions are heavily left-skewed and the BPVS score distribution is right-skewed.

A salient feature of the children’s distribution is the disproportionately small number of C3 responses; a potential limitation of the question as elicited. As such, a secondary analysis reports agreement between affirmative responses (C4 and C5) vs. non-affirmative responses (C1, C2 and C3) for children, and for mothers (M4-M5 vs. M1-M3) and teachers (T4-T5 vs. T1-T3).

### Pairwise agreement between assessments of academic performance

Table 2 presents the pairwise distributions of academic performance perceptions between child-mother (n = 870), child-teacher (n = 531), and mother-teacher (n = 545) participants.

**Table 2:** Pairwise distributions of academic performance perceptions between child-mother (n = 870), child-teacher (n = 531), and mother-teacher (n = 545) assessment pairs.

	n	(%)	n	(%)	n	(%)	n	(%)	n	(%)
	Mother									
Child	M1		M2		M3		M4		M5	
C1	0	(0.0)	0	(0.0)	2	(0.2)	5	(0.6)	3	(0.3)
C2	0	(0.0)	0	(0.0)	9	(1.0)	18	(2.1)	17	(2.0)
C3	0	(0.0)	0	(0.0)	0	(0.0)	0	(0.0)	1	(0.1)
C4	1	(0.1)	3	(0.3)	55	(6.3)	143	(16.4)	117	(13.4)
C5	0	(0.0)	3	(0.3)	68	(7.8)	243	(27.9)	182	(20.9)
	Teacher									
Child	T1		T2		T3		T4		T5	
C1	0	(0.0)	3	(0.6)	1	(0.2)	0	(0.0)	0	(0.0)
C2	5	(0.9)	4	(0.8)	10	(1.9)	4	(0.8)	1	(0.2)
C3	0	(0.0)	0	(0.0)	0	(0.0)	1	(0.2)	0	(0.0)
C4	15	(2.8)	42	(7.9)	75	(14.1)	44	(8.3)	11	(2.1)
C5	24	(4.5)	68	(12.8)	115	(21.7)	82	(15.4)	26	(4.9)
	Teacher									
Mother	T1		T2		T3		T4		T5	
M1	0	(0.0)	0	(0.0)	0	(0.0)	1	(0.2)	0	(0.0)
M2	0	(0.0)	0	(0.0)	2	(0.4)	1	(0.2)	0	(0.0)
M3	9	(1.7)	30	(5.5)	28	(5.1)	13	(2.4)	0	(0.0)
M4	34	(6.2)	53	(9.7)	106	(19.4)	55	(10.1)	13	(2.4)
M5	6	(1.1)	37	(6.8)	69	(12.7)	62	(11.4)	26	(4.8)

C1, M1 and T1 are the lowest perceived ratings for children, mothers and teachers respectively, whereas C5, M5 and T5 are the highest perceived ratings.

#### Child-mother assessment pairs

Both the test for symmetry (p<0.001) and marginal homogeneity (p<0.001) were significant, underlining the distributional differences observed in Fig 1. Children’s self-assessments were more frequently higher than their mothers than vice versa. The pairwise agreement estimate was κ = 0.03 (95% CI: -0.03, 0.09), representing negligible agreement beyond chance. When dichotomising the responses, significant asymmetry and marginal heterogeneity remained (both p<0.001) and agreement continued to be negligible (κ = 0.02; 95% CI: -0.04, 0.09).

#### Child-teacher assessment pairs

Again, both the symmetry (p<0.001) and marginal homogeneity tests (p<0.001) were significantly different between assessment pairs. Here, children’s self-assessments were more frequently higher than their teachers’ global assessments than vice versa. The estimated κ was 0.04 (95% CI: 0.01, 0.08), which indicates negligible agreement beyond chance. Significant asymmetry and marginal heterogeneity remained (both p<0.001) when responses were dichotomised, and agreement did not improve (κ = 0.02; 95% CI: -0.01, 0.04).

#### Mother-teacher assessment pairs

The symmetry (p<0.001) and marginal homogeneity tests (p<0.001) were significant for the mother-teacher assessment pairs. Mothers’ assessments were more frequently higher than the teachers’ global assessments. The estimated κ was 0.11 (95% CI: 0.07, 0.15), which represents only slight agreement. Similarly, significant asymmetry and marginal heterogeneity remained when responses were dichotomised (both p<0.001), and estimated agreement was negligible (κ = 0.07; 95% CI: 0.03, 0.11).

#### Teacher oral language assessment against the BPVS direct measure

**Fig 2:**
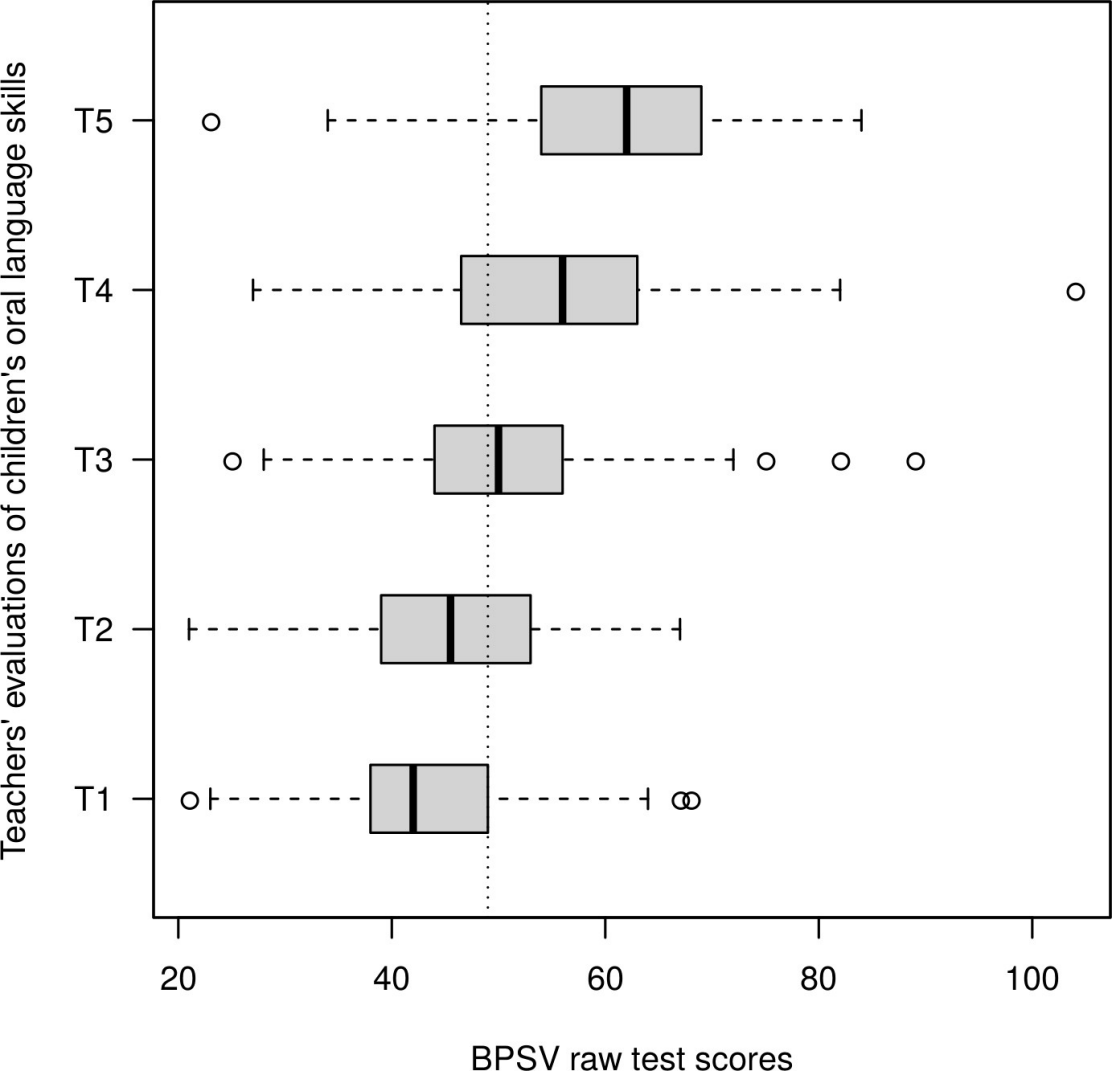
Teachers’ assessments of children’s oral language skills and the BPVS raw scores. The dotted line (the BPVS raw score of 49) indicates the median for the whole group.

Teachers’ assessments of children’s oral language skills were plotted against the children’s raw test scores from the BPVS in Fig 2. A higher median BPVS raw score was observed for each level increase in teacher assessment ratings; an observation supported by ordinal logistic regression whereby the estimated odds of receiving a higher teacher rating increased by 1.08 (95% CI: 1.06, 1.09) for each unit increase in the BPVS raw scores. These odds remained significant when adjusted for household income, teachers’ sex, ethnicity and experience, and children’s sex and ethnicity; estimated as 1.08 (95% CI: 1.06, 1.10). The estimate for intraclass correlation coefficient in the BPVS scores within each teacher rating was 0.22 (95% CI: 0.08, 0.70).

### Sensitivity of results to the exclusion of mathematics domain

All concordance and agreement statistics were replicated when the global teacher assessment scores were computed without the mathematics domain. The largest absolute effect size difference detected across all κ statistics being 0.02 for mother-teacher assessment pairs when this global teacher assessment scores with the mathematics estimate was compared to the global score without mathematics estimate.

### Participant characteristics associated with concordance patterns

Given teacher’s training and experience, the marginal distributions presented in Fig 1, and the significant increasing association between teachers’ oral language assessment of children against their BPVS direct measure, the teacher’s global assessment of the children’s academic performance was treated here as a reference standard in these secondary analyses. Child-teacher and mother-teacher assessments were each investigated, comparing those who yielded concordant (i.e. pairs with the same rating, such as [C5 and T5]) or nearly concordant assessments (i.e. pairs differing by one level, such as [C5 and T4] or [T5 and C4]) to those who were discordant and had a substantially greater academic performance perception than their teacher (i.e. [C5 and T1], [C5 and T2] and [C4 and T1] for the child-teacher comparison, and [M5 and T1], [M5 and T2], and [M4 and T1] for the mother-teacher comparison). Overall, there were 261 and 335 concordant or nearly concordant (C) response pairs for the child-teacher and mother-teacher assessments, respectively; and 107 and 77 discordant and greater academic performance perception than teacher (D) responses. The characteristics of study participants partitioned by these C and D classifications appear in Table 3.

**Table 3:** Distribution of characteristics of child-teacher and mother-teacher responses for those with complete or near concordance (C) compared to those who were discordant and had a substantially greater academic performance perception than the teacher (D).

		Child-teacher				Mother-teacher			
		C		D		C		D	
		n	(%)	n	(%) p-value	n	(%)	n	(%) p-value
**Child characteristics**									
*Sex*					0.82				0.70
	Female	125	(47.9)	53	(49.5)	155	(46.3)	38	(49.4)
	Male	136	(52.1)	54	(50.5)	180	(53.7)	39	(50.6)
*Ethnic identification*					0.58				0.25
	Samoan	117	(49.8)	54	(53.5)	141	(46.7)	44	(59.5)
	Tongan	42	(17.9)	12	(11.9)	58	(19.2)	13	(17.6)
	Cook Islands Māori	13	(5.5)	5	(5.0)	19	(6.3)	3	(4.1)
	Other Pacific	63	(26.8)	30	(29.7)	84	(27.8)	14	(18.9)
*CBCL internalising behaviour*					0.19				0.39
	Normal range	206	(78.9)	92	(86.0)	272	(81.2)	61	(79.2)
	Borderline	25	(9.6)	9	(8.4)	28	(8.4)	10	(13.0)
	Clinical	30	(11.5)	6	(5.6)	35	(10.4)	6	(7.8)
*CBCL externalising behaviour*					0.33				0.10
	Normal range	174	(66.7)	79	(73.8)	218	(65.1)	58	(75.3)
	Borderline	40	(15.3)	15	(14.0)	54	(16.1)	12	(15.6)
	Clinical	47	(18.0)	13	(12.1)	63	(18.8)	7	(9.1)
*BPVS standard score*					<0.001				<0.001
	Below norm	8	(3.1)	17	(15.9)	9	(2.7)	9	(12.3)
	At norm	212	(81.2)	88	(82.2)	281	(85.4)	64	(87.7)
	Above norm	41	(15.7)	2	(1.9)	39	(11.9)	0	(0.0)
**Maternal characteristics at baseline**									
*Age at childbirth (years)*					0.12				0.15
	<20	17	(6.5)	10	(9.3)	23	(6.9)	7	(9.1)
	20–24	55	(21.1)	24	(22.4)	70	(20.9)	20	(26.0)
	25–29	82	(31.4)	24	(22.4)	107	(31.9)	13	(16.9)
	30–34	64	(24.5)	29	(27.1)	79	(23.6)	23	(29.9)
	35–39	37	(14.2)	12	(11.2)	44	(13.1)	11	(14.3)
	≥40	6	(2.3)	8	(7.5)	12	(3.6)	3	(3.9)
*Ethnic identification*					0.21				0.16
	Samoan	117	(44.8)	54	(50.5)	141	(42.1)	44	(57.1)
	Tongan	42	(16.1)	12	(11.2)	58	(17.3)	13	(16.9)
	Cook Islands Māori	63	(24.1)	30	(28.0)	84	(25.1)	14	(18.2)
	Other Pacific	14	(5.4)	7	(6.5)	23	(6.9)	3	(3.9)
	Non-Pacific	25	(9.6)	4	(3.7)	29	(8.7)	3	(3.9)
*Highest educational qualification*					0.004				<0.001
	No formal qual.	68	(26.1)	41	(38.3)	98	(29.3)	33	(42.9)
	Secondary	102	(39.1)	46	(43.0)	126	(37.6)	34	(44.2)
	Post-secondary	91	(34.9)	20	(18.7)	111	(33.1)	10	(13.0)
*Parity*					0.07				0.27
	1	64	(24.8)	22	(21.2)	75	(22.7)	16	(21.1)
	2–4	160	(62.0)	58	(55.8)	210	(63.6)	44	(57.9)
	≥5	34	(13.2)	24	(23.1)	45	(13.6)	16	(21.1)
*Relationship status*					0.46				0.99
	Married/de facto	210	(80.5)	90	(84.1)	277	(82.7)	64	(83.1)
	Single	51	(19.5)	17	(15.9)	58	(17.3)	13	(16.9)
*English fluency*					<0.001				0.03
	Proficient	184	(70.5)	55	(51.4)	223	(66.6)	41	(53.2)
	Otherwise	77	(29.5)	52	(48.6)	112	(33.4)	36	(46.8)
*Cultural orientation*					0.04				0.11
	Integration	58	(22.5)	20	(18.9)	61	(18.4)	19	(25.3)
	Assimilation	101	(39.1)	29	(27.4)	121	(36.4)	17	(22.7)
	Separation	63	(24.4)	40	(37.7)	94	(28.3)	26	(34.7)
	Marginalisation	36	(14.0)	17	(16.0)	56	(16.9)	13	(17.3)
*Household income (NZD)*					0.78				0.54
	≤$20,000	81	(31.0)	34	(31.8)	104	(31.0)	23	(29.9)
	$20,001-$40,000	136	(52.1)	53	(49.5)	178	(53.1)	44	(57.1)
	>40,000	35	(13.4)	14	(13.1)	42	(12.5)	6	(7.8)
	Unknown/declined	9	(3.4)	6	(5.6)	11	(3.3)	4	(5.2)
**Teacher characteristics at 6-years**									
*Sex*					0.99				0.57
	Female	241	(93.1)	99	(93.4)	317	(94.9)	69	(93.2)
	Male	18	(6.9)	7	(6.6)	17	(5.1)	5	(6.8)
*Ethnic identification*					0.01				0.03
	NZ European/Pākehā	117	(45.5)	50	(48.1)	164	(49.4)	36	(49.3)
	Pacific	53	(20.6)	18	(17.3)	66	(19.9)	8	(11.0)
	Māori	4	(1.6)	9	(8.7)	9	(2.7)	7	(9.6)
	Other	83	(32.3)	27	(26.0)	93	(28.0)	22	(30.1)
*Years of teaching experience*					0.03				0.05
	0–5	117	(46.1)	48	(47.1)	156	(47.6)	36	(48.6)
	6–10	72	(28.3)	17	(16.7)	87	(26.5)	11	(14.9)
	>10	65	(25.6)	37	(36.3)	85	(25.9)	27	(36.5)

p-values derived from Fisher’s exact test.

Among the child-teacher responses, significantly higher proportions of children in C pairs were found to score above the population norm on the BPVS test (p<0.001); have mothers with higher educational qualifications (p = 0.004), English proficiency (p<0.001), assimilated or integrated cultural orientation (p = 0.04); and have teachers with 6–10 years of teaching experience (p = 0.03), and who were of Pacific ethnicity (p = 0.01). For the mother-teacher responses, significantly higher proportions of mothers in C pairs were found to have post-secondary educational qualifications (p<0.001), be proficient in English (p = 0.03), and have children who performed above the population norm on the BPVS test (p<0.001). A significantly higher proportion of teachers in these C pairs were found to be of Pacific ethnicity (p = 0.03).

Given the association between maternal cultural orientation and the observed concordance patterns for child-teacher pairs in Table 3, further analyses were conducted to examine if this relationship could have been confounded by other maternal characteristics. Accordingly, all the maternal characteristic variables referred to in Table 3 that were significantly associated with concordance patterns were analysed. In particular, maternal education was significantly associated with the concordance patterns and hence considered as a potential confounder. Although maternal education was significantly associated with maternal cultural orientation (p<0.001), the cultural orientation measure remained significant when both were included in a model for concordance. Maternal English language fluency was not separately analysed as the cultural orientation measure included questions on preferred language use in social situations and language was treated as part of one’s cultural and social profile [15].

## Discussion

Important asymmetrical differences in the perceptions of academic performance held by Pacific children, mothers, and teachers were identified. Children and mothers were significantly more likely to rate Pacific children’s academic performance highly than their teachers. Moreover, there was negligible to slight agreement beyond chance found between any of the pairwise investigations. This implies that some mothers’ and Pacific children’s perceptions are likely to be in stark contrast to their teachers. Results from the ordinal logistic regression demonstrated that the teachers provided oral language evaluations that significantly and consistently increased with the children’s increasing BPVS scores. If teachers’ assessments more closely align to the criteria used for established and reported performance measures, then this child and maternal discordance in perceptions may contribute to New Zealand’s relatively large international literacy and numeracy disparity, and over-representation of Pacific children [6, 7].

The cohort children’s generally positive academic self-perceptions at age 6 years are consistent with the results found in earlier literature on children’s academic self-concept [49–52]. However, the results from the additional analyses linking the concordance patterns to environmental factors in the present study confirmed the association between children’s strong English receptive vocabulary skills and the concordance between their academic self-perception and the achievement level assessed by their teachers. On the other hand, the children who were assessed by their teachers as not performing as strongly as their peers were somehow protected from developing negative academic self-perception as indicated by the optimistic self-assessment of these children. Considering the positive association between academic self-perception and achievement [26–28], this in itself may not be a deleterious outcome. However, the results nevertheless suggest that the communication between home and school about children’s school performance may not have been completely effective. The language may have been a barrier to some Pacific mothers considering that a higher proportion of them reporting lower proficiency in the English language were rating their children’s performance more favourably compared to the teacher’s assessment. The premise that there may have been potential issues with communication is also more convincing given that there was a higher degree of agreement between teachers and mothers who had higher educational qualifications. Indeed, in a study of 90 Pacific parents, low self-esteem, lack of fluency in the English language and the lack of understanding about the New Zealand educational context were expressed as being the key reasons for Pacific parents’ “culture of silence” [53] during educational consultations. Providing culturally appropriate support for Pacific families, for example, using Pacific languages for communication and having Pacific staff members who can help students and families navigate the New Zealand educational system, was noted as a way to empower Pacific students and parents [8, 12, 53–55].

Such mitigating actions are called for as explicit parent-teacher conflicts can often result from disagreement on the perceptions of children’s ability [56]. Patel and Stevens, in their 2010 study of 179 American sixth to eighth graders (aged 11 to 14 years), also found discrepancies in the perceptions of children’s academic abilities between parents, teachers, and students [57]. Children’s self-perceptions of general scholastic abilities were compared to their parents’ perceptions of their children’s abilities in mathematics and English/language arts subjects and the actual grades given by the teachers. The results of bivariate linear regressions revealed that in general, as parent-student or parent-teacher discrepancies in perceptions of students’ academic abilities increased, the parents tended to participate less in school activities and the schools facilitated less opportunities for parents to get involved. These results support the argument that the family-school interactions are importantly shaped by the ideas and perceptions held by students, parents and teachers [57–59]. Establishing joint understanding of perceptions of children’s scholastic abilities could be an important step toward enhanced home-school collaborations and parental involvement in children’s education [57, 59].

However, here in the context of Pacific students and families, the disagreement in perceptions is unlikely to be explicit or shared between individuals [12, 53]. Most Pacific cultures emphasise the importance of deference towards authority figures, the teachers in this instance, which may result in Pacific students and their parents not questioning teachers’ views or teaching methods employed [12, 14, 53]. It is also possible that Pacific mothers have more holistic and culturally connected conception of educational success than the teachers in the English-medium schools [8, 12, 54]. A shared communicated understanding of the expectations for academic achievement is vital as is the mutual respect for ethnic and cultural differences [53–55]. Maintaining strong links to their Pacific cultural values and communities has been shown to have positive impact on health among new mothers in New Zealand [15], and it could be opined that such strong cultural connections would also lead to better educational and wellbeing outcomes for Pacific children who remain outside the dominant New Zealand European/Pākehā culture.

Pacific families especially stand to gain from strategies that help reduce barriers to effective communication and home-school collaboration in promoting educational success among Pacific students [53, 60]. The result showing the largely discordant perceptions of academic performance held between Pacific mothers and teachers is of more import considering that the difference in views may be left unsaid and unaddressed [12, 53]. In efforts to encourage dialogues between Pacific families and schools, providing a culturally inclusive environment that removes power imbalance between the dominant New Zealand and Pacific cultures is often recommended as well as the use of Pacific languages [8, 12, 53–55]. Rendering the classroom and school environment more linguistically inclusive by having visible signs in students’ first languages is also found to help families from ethnic minority backgrounds feel welcomed at school [61, 62]. Providing support to Pacific students and families by employing Pacific personnel to facilitate communication has been found effective in some studies [8, 12]. The results found in the current study emphasise the need to enhance home-school communication, and encourage Pacific parents’ involvement at school by fostering inclusiveness and expressing cultural responsiveness towards Pacific families [8, 53–55].

For children aged 6 years in New Zealand, the teacher’s overall judgement of a student’s scholastic progress often forms the basis for the student’s academic achievement outcomes, which, until 2018, was regularly compared against national standards [63]. Even with the practice of benchmarking each student against predetermined achievement standards, there is still a potential for bias in teachers’ assessments. For example, in a 2016 study involving 519 American fifth and sixth graders, Mowrey and Farran found that the teachers in the study tended to recalibrate their students’ performances in mathematics based on the students within their school rather than to the national norms [64]. This had the effect of inflated ratings for culturally diverse students from lower socioeconomic backgrounds. An attempt has been made in this study to gain insight into this phenomenon by comparing teachers’ assessments with the BPVS scores. The results from the ordinal logistic regression showed that the teachers in this study provided evaluations that tended to increase monotonely as the children’s BPVS raw test scores increased. This result validated the teacher assessment of children’s oral language development in a sense that the teacher assessments resembled the distribution of a language assessment tool that is based on externalised and directly measurable standards. However, the coefficient estimate for intraclass correlation in the BPVS raw scores within each grade showed relatively poor agreement. The presence of unusual observations could have influenced the analyses of squared deviations in the computation of intraclass correlation coefficients. The estimated confidence interval was indeed wide in the current result. The analyses of median and the interquartile ranges were reported to supplement the result of the correlation estimate. The median scores, which are not strongly influenced by unusual observations, show a clearer trend of monotone increases in the raw scores for each increase in the grades given by the teachers.

Some commonly held reasons for discrepancies in multi-informant responses are relatively strong within-observer effects that are often stronger than the inter-observer effects [18, 65, 66] and children’s behaviours being situation-specific [23, 25, 67]. Of these, the relatively strong within-observer associations have been observed in studies across various domains of children’s behavioural studies. For example, it has been noted that the lack of agreement between parent and teacher dyads on their observations of children’s behaviours may be due to dealing with children within disparate settings and environments [23, 66, 67]. Children are thought to display a different range of emotions and behaviours depending on the situation and the setting, hence allowing parents and teachers to observe children’s behaviours within the context of home and school [25, 66, 67]. It is also noted that parents and teachers may exhibit systematic discrepancies in their observations as they may have different benchmarks to compare a child to (for example, a sibling at home or other students in class), and also have varying degrees of tolerance if a child displays any problematic behaviours [24, 68].

The difference in the benchmark used by the teachers and Pacific mothers could have influenced the results here. Miller and Davis have noted that one of the general findings from studies that compared mothers’ assessments of their children’s performances with those of the teachers’ or the results from standardised tests among children with LDs was the tendency of mothers to overestimate the children’s performances [69]. Their own study of 60 American second and fifth graders without LDs indicated that, although both mothers and teachers tended to overestimate children’s cognitive abilities, the teachers tended to be more accurate in terms of students’ relative standing within their class. The mothers in the present study perceived their children’s school performance more positively than the teachers. The teacher assessment tended to concord with the performance measured using the standardised test implying that if the standardised assessments are taken as the gold standard for judging the accuracy of an assessment, then this could lead to disaccord between parental perceptions and the documented educational outcomes. Pacific educators, parents and communities have voiced their sense of disempowerment at the continued reports of disparity in educational outcomes between Pacific students and their peers [53]. Pacific people in New Zealand have also expressed their yearning to be successful in both the New Zealand and Pacific contexts without compromising their cherished traditions and cultural values [70]. In order to help them achieve this, the support provided to Pacific students must be culturally responsive as younger generations of Pacific people can struggle with their cultural identities and acculturation [71, 72]. Standardised assessments may provide useful information about student performance and perhaps it can be used in addition to the measures of culture-connected ways of learning often internalised by the indigenous Māori and Pacific learners [73].

This study had a number of strengths, including the large data size of a policy priority population, the measurement wave timing at 6-years and the availability of multiple apposite variables, which were pre-assessed for its cultural appropriateness through consultations with Pacific stakeholders and pilot-testing [11, 15]. Moreover, the impact of maternal cultural orientation on child-teacher or parent-teacher concordances, to our knowledge, has not been studied previously. This study also deliberately and explicitly includes the children’s perspectives. The primary basis often cited by researchers for excluding children’s perspectives in their studies is the lack of reliability and validity in children’s self-reported measures [29]. However, a 2008 study of child-teacher relationships among ethnic minority children in urban kindergarten classrooms indicated meaningful differences between perceptions held by the children and their teachers [29]. They demonstrated that these differences enhance the understanding of the child-teacher relationships and, more importantly, help to identify problems with over-relying on teacher’s reports alone. These results, in addition to very disparate perceptions of their own academic performance held by the Pacific children compared to that of the mothers and teachers, warrant further research into aligning and explaining the differences in student’s and teacher’s assessments.

The study is not without its limitations. The cohort of Pacific children was deliberately recruited from the wider Auckland region, New Zealand’s most populated city, where almost two thirds of New Zealand’s Pacific people reside [74]. Moreover, the cohort was drawn from the population born in year 2000 and some of the results may not be applicable to more recent populations. As such caution should be exercised when generalising to contemporary Pacific communities elsewhere in New Zealand. Replicating the main findings of this study using more recent data could improve generalisability of the results. The questions put to the participants were not identical, mainly so that they were developmentally appropriate for children, which nevertheless may have introduced unmeasured systematic biases. Also, unlike the teacher assessments, the child and maternal questions were generic and lacked subject-specificity. Ideally each respondent would have been questioned identically on each educational domain of interest. However, the clear differences in self-reported assessments remain notable and important.

This study analysed and reported the concordant and discordant group differences in various sociodemographic factors. The observed differences in maternal education, cultural orientation and language fluency warrant further investigation into how these variables may interact and influence concordance patterns. The analyses also considered the potential confounding effects of maternal education on the association between maternal cultural orientation and the child-teacher concordance. The results demonstrated that the maternal cultural orientation was related to the proportion of children who were in concordance with their teachers on their schoolwork performance even after accounting for the differences in the mother’s educational background. Based on the current measures utilised and analyses employed, the effects of English language fluency could not be disentangled from those of maternal cultural orientation. However, as many socialisation and socio-cultural studies attest, language is closely connected to and can manifest as a mediating tool in acquisition of one’s cultural knowledge [75, 76]. Indeed, the history of Pacific migration to New Zealand and the resulting acculturation process go hand in hand with the loss of community languages among Pacific migrants [77–80]. The observed disparity in perceptions held about Pacific children’s academic performance may have risen within this sociocultural context of English language being regarded as the mainstay of academic learning in the majority of New Zealand schools. It is unclear whether it would be culturally appropriate to separate languages from the cultural competence construct, as the measures used here were assessed by the Pacific Advisory Team as fitting cultural expectations [11, 15]. However, one way to examine these constructs on their own is to exclude the language related items from the cultural orientation measure and consider its relationship with the concordance patterns. Such analyses will be able to confirm whether language proficiency has any differential impact on the concordance profiles compared to that of other cultural artefacts and manifestations.

As with all studies using self-reported measures, various sources of bias may impact on the findings. Not all eligible PIF study children had complete information from their teachers and parents. Such missing observations could compromise internal validity if the missing information importantly differs from those of the observed. Notable here was that mothers without any formal qualifications were more likely to have a child without a teacher’s assessment. Moreover, these mothers were more likely to have perceptions that were discordant and optimistic compared to the teachers. Thus the underlying levels of mother-teacher discordance presented here is likely to be an underestimate. Lastly, the BPVS test, like most other direct measures designed for a monolingual population, is likely to suffer from cultural bias if tested on an English-as-additional-language population [81, 82] or in other geographic locations. However, all PIF measures and assessment tools were reviewed by the Pacific scientific advisory board for their cultural appropriateness and successfully piloted prior to the actual use in the questionnaires [11, 15]. While this provides some face validity to the measures used here, the analyses would have been strengthened if other standardised measures were available for corroboration and sensitivity check.

Despite the noted limitations, this study provided novel empirical evidence for the generally optimistic perceptions of academic achievement held between Pacific children aged 6 years and their mothers compared to their teachers in English-language medium schools. Having described the differences in the level of concordance and the characteristics between concordant and discordant pairs, it behoves future investigation into a more nuanced and comprehensive study that specifically aims to analyse these characteristics as predictors for concordance outcomes. In this study, an implicit assumption was made about the underlying culture of school; that it is predominantly Eurocentric monoculturalism. However, schools in New Zealand, like the families studied here, are not homogeneous, and important exceptions to this assumption exist such as bilingual immersion programmes and community-focused campuses [77, 83]. Unlike the maternal cultural orientation, the diversity in school cultures was not explicitly studied here. However, this remains an important area for further exploration, which would add valuable insight into how the interaction between cultures influence our perceptions of academic success. Colonialization is an ongoing process, and the socio-political context outside the school environment can also influence educational outcomes [84]. Future studies should also look at how historical trauma and systematic discrimination can lead to the perpetuation of inequity in educational and health outcomes among Pacific people in New Zealand.

## Conclusion

This study presented results from the analyses of agreement and concordance between Pacific children and their mothers and teachers on the children’s schoolwork performance in the first year of school. The main findings of the study suggest that different perceptions of schoolwork performance were held by these groups of participants, and that these differences may relate to the families’ social and cultural background. Compared to the teacher assessment, the children in this study expressed more optimistic views of their academic progress. Without timely feedback on schoolwork performance, many Pacific children are at risk of being left with these disparate perceptions longer and further lagging behind their peers [60]. English literacy intervention studies involving smaller samples of Pacific students suggest the efficacy of early, targeted interventions [85, 86]. Efforts to align the student perceptions with documented achievement outcomes could include effective and timely feedback on academic progress, redefining how we measure children’s progress at school, and instigating culturally appropriate strategies and interventions to prevent Pacific children from being left behind educationally, and ultimately compromising their future opportunities, health and wellbeing.

This study challenged the culture-free assumption about schools in New Zealand and analysed the perceptions of schoolwork performance from multiple perspectives–those of Pacific children, mothers and teachers. The main findings of this study reaffirm the importance of enhancing communication between home and school, and teachers demonstrating cultural responsiveness towards Pacific students and their families [87]. Dismantling linguistic and cultural barriers experienced by Pacific mothers largely “separated” from the New Zealand culture could encourage them to initiate conversations with teachers on their children’s academic progress and be more involved at school. For example, the initial teacher education could equip teachers with the knowledge of Pacific perspectives and values by incorporating them into teacher preparation programmes and materials [55, 88]. The concept of assessment can also be broadened to include more holistic measures that are culturally appropriate and cater for the needs of a diverse and multilingual student body [73]. Such demonstration of Pacific cultural values by teachers and schools could help improve home-school continuity for migrant Pacific children and enhance their educational experience. Providing support for those families at risk of cultural separation and marginalisation, and preparing teachers to be culturally responsive are important policy implications from this study.

## Supporting information

S1 Table Additional information on the standardised measure. (DOC)

S2 Table Additional information on selected variables. (DOC)
