# Supplementary material for: Perceptions of Pacific children’s academic performance at age 6 years: A multi-informant agreement study
Source: PLoS One. 2020 Oct 16;15(10):e0240901. doi: 10.1371/journal.pone.0240901 (PMC7567394; doi:10.1371/journal.pone.0240901)
Supplement: S1 Table — (DOC) [file pone.0240901.s001.doc]

# S1. Table. Additional information on the standardised measure.

| **Standardised measure** | **Description** |
| --- | --- |
| British Picture Vocabulary Scale (BPVS) | The BPVS is a one-to-one test measuring children's receptive vocabulary skills and is suitable for pre-school to secondary school students. The test comprises 14 sets of 12 items. Each item has four black and white pictures and children can select the picture that they think best represents the word read out or explained to them. Children usually start at the age-appropriate set and work down until a basal set (a set for which a maximum of 2 errors are permitted) is established. From this basal set, they work upwards until they reach their ceiling set, a set where 8 or more errors are made. Raw scores obtained from the test can be translated into population standardised scores and compared to the age-equivalents [1-4]. Standardised scores are useful when a comparison needs to be made to a large, nationally representative sample that have previously taken the test [4]. Standardised scores between 85 and 115 indicate the 'age-expected' range. Based on split-half reliability tests, median reliability coefficients of 0.80 for 3 to 7 year olds and 0.81 for 4 to 9 year olds have been reported [2]. |

| [1] Atkinson L. The British Picture Vocabulary Scale: constructing confidence intervals to evaluate change. Br. J. Disord. Comm. 1991;26:369-72. |
| --- |
| [2] Gathercole S, Willis C, Baddeley A, Emslie H. The child's test of nonword repetition: a test of phonological working memory. Memory. 1994;2:103-27. |
| [3] Mahon M, Crutchley A. Performance of typically-developing school-age children with English as an additional language on the British Picture Vocabulary Scale II. Child Lang. Teach. Ther. 2006;22:333-51. |
| [4] Thornley H. An investigation into the knowledge of receptive vocabulary in a first and second language. A case study on Dizygotic twins. Innervate. 2010;3:453-79. |
