# Supplementary material for: Perceptions of Pacific children’s academic performance at age 6 years: A multi-informant agreement study
Source: PLoS One. 2020 Oct 16;15(10):e0240901. doi: 10.1371/journal.pone.0240901 (PMC7567394; doi:10.1371/journal.pone.0240901)
Supplement: S2 Table — (DOC) [file pone.0240901.s002.doc]

# S2. Table. Additional information on selected variables.

| **Variables** | **Description** |
| --- | --- |
| **Baseline sociodemographic variables:** | |
| Maternal age | Maternal age was determined from the difference in years between the date of birth and the date of interview. The number of years thus obtained was then categorised into either less than 20 years; 20-24 years; 25-29 years; 30-34 years; 35-39 years; and 40 plus years. |
| Maternal ethnicity | Mothers selected an ethnic group that they identified most with from the following options: Samoan; Cook Islands; Niuean; Tongan; Fijian; Fijian Indian; Tokelauan; Tuvaluan; Māori; New Zealand European/Pākehā; and Other. These responses were further collapsed into Samoan; Cook Islands; Tongan; Other Pacific – to include those Pacific Islands other than the previous three – and Non-Pacific Island – to include those mothers who were eligible due to their Pacific partners. |
| Maternal acculturation (General Ethnicity Questionnaire - GEQ) | Mothers’ acculturation characterisations were categorised into four types: Assimilator; Separatist; Integrator and Marginalisor. These characterisations were made at the baseline (6-weeks postpartum) using a modified GEQ [1-3]. The original 38-item GEQ scale was reduced to 11 items to minimise burden on respondents. The modified versions focused on 1) the maintenance of one's heritage, culture and identity and 2) one's preference for connecting and participating in the larger society. Modifications were also made to exclude items that have limited relevance to New Zealand and add items specific to Pacific culture such as social affiliations and participation in Pacific sports and recreational activities [4]. Cronbach’s α of 0.81 and 0.83 have been reported for NZ and Pacific acculturation measures in this cohort [4]. |
| Maternal education | Mothers’ highest educational qualifications were elicited from several questions. At the baseline, they were asked about their 1) highest school qualification (No formal qualification; New Zealand School Certificate in 1 or more subjects; New Zealand 6th Form Certificate in 1 or more subjects; New Zealand University Entrance (pre 1986) in 1 or more subjects; New Zealand Higher School Certificate or Higher Leaving Certificate; New Zealand University Bursary or Scholarship; Other New Zealand secondary school qualification; Overseas secondary school qualification; Don’t know, decline; and Missing), and their 2) highest post-school qualification (No other qualification; Trade Certificate; Advanced Trade Certificate; New Zealand Certificate or Diploma; Technicians Certificate; Polytechnic Certificate or Diploma; Teachers Certificate or Diploma; Other; Don’t know, decline; and Missing). The responses to the above questions were recoded into a new variable with three category responses: No formal qualification; Secondary; Post-secondary qualification. |
| **Children’s behavioural development:** | |
| Child Behaviour Checklist (CBCL) | Parental/caregiver ratings of behavioural/emotional problem behaviours were assessed at 6-years using the 120-item Child Behaviour Checklist for ages 6-18 (CBCL/6-18) year groups [5, 6]. The CBCL problem behaviours are largely separated into internalising and externalising behaviours. The scales are normed according to age and gender categories for each syndrome. For each syndrome, the clinical cut-off corresponded to above 98th percentile and the borderline to 95th-98th percentile. For composite scales (internalising and externalising), clinical cut-off corresponded to 90th percentile and the borderline 83rd. |
| CBCL-Internalising behaviour | For CBCL/6-18, the score for the internalising behaviour problems is the sum of scores for 32 questions within three syndromes: anxious/depressed, withdrawn, and somatic complaints. |
| CBCL-Externalising behaviour | The score for externalising behaviour is the sum of scores for 35 questions within two syndromes: aggression and rule-breaking. |

[1]. Berry JW. Conceptual approaches to acculturation. In: Chun KM, Organista PB, Gerardo M, editors. Acculturation: advances in theory, measurement, and applied research. Washington (DC): American Psychological Association; 2003. p. 17-38.

[2]. Berry JW. Contexts of acculturation. In: Davis LS, Berry JW, editors. The Cambridge handbook of acculturation psychology. Cambridge (UK): Cambridge University Press; 2006. p. 27-42.

[3]. Tsai JL, Ying YW, Lee PA. The meaning of ‘being Chinese’ and ‘being American’: variation among Chinese American young adults. J. Cross Cult. Psychol. 2000;31(3):302-22.

[4]. Borrows J, Williams M, Schluter PJ, Paterson J, Helu S. Pacific Islands Families Study: the association of infant health risk indicators and acculturation of Pacific Island mothers living in New Zealand. J. Cross Cult. Psychol. 2010;42(5):699-724.

[5]. Paterson J, Taylor S, Schluter PJ, Iusitini L. Pacific Islands Families (PIF) Study: behavioural problems during childhood. J. Child Fam. Stud. 2013;22:231-43.

[6]. Achenbach T, McConaughy S, Ivanova M, Rescorla L. Manual for the ASEBA Brief Problem Monitor. Burlington (VT): Research Center for Children, Youth and Families, University of Vermont; 2011.
